# Xylene (all isomers) – Addendum: re-evaluation of the BAT value and of the pregnancy risk group for the BAT value: Assessment Values in Biological Material – Translation of the German version from 2025

**DOI:** 10.34865/bb133020e10_1ad

**Published:** 2025-03-31

**Authors:** Thomas Jäger, Wobbeke Weistenhöfer, Hans Drexler, Andrea Hartwig, MAK Commission

**Affiliations:** 1 BASF SE. Corporate Health Management Carl-Bosch-Straße 38 67056 Ludwigshafen Germany; 2 Friedrich-Alexander-Universität Erlangen-Nürnberg. Institute and Outpatient Clinic of Occupational, Social, and Environmental Medicine Henkestraße 9–11 91054 Erlangen Germany; 3 Institute of Applied Biosciences. Department of Food Chemistry and Toxicology. Karlsruhe Institute of Technology (KIT) Adenauerring 20a, Building 50.41 76131 Karlsruhe Germany; 4 Permanent Senate Commission for the Investigation of Health Hazards of Chemical Compounds in the Work Area. Deutsche Forschungsgemeinschaft, Kennedyallee 40, 53175 Bonn, Germany. Further information: Permanent Senate Commission for the Investigation of Health Hazards of Chemical Compounds in the Work Area | DFG

**Keywords:** xylene (all isomers), methylhippuric acid, biological tolerance value, BAT value, developmental toxicity

## Abstract

The German Senate Commission for the Investigation of Health Hazards of Chemical Compounds in the Work Area (MAK Commission) summarised and re-evaluated the data for the biological tolerance value (BAT value) for xylene [1330-20-7] after the occupational exposure limit value (maximum concentration at the workplace, MAK value) for this substance had been lowered from 100 to 50 ml/m^3^ taking into account the increased respiratory volume in the workplace. Since the last evaluation, new publications have been identified from a literature search and relevant studies are described in detail. For the derivation of the BAT value, both occupational field studies and experimental studies showed a good correlation between the xylene concentration in the air and the methylhippuric acid concentration in the urine. Considering the available studies and an increased respiratory volume for experimental studies without physical excercise, an average of 1800 mg methylhippuric acid/g creatinine can be expected after eight-hour exposure to xylene (all isomers) at the level of the MAK value and was therefore established as the BAT value. Sampling should take place at the end of exposure or the end of a shift. In 2021, the MAK value for xylene was re-evaluated with regard to developmental toxicity. No comprehensive studies on developmental neurotoxicity were available, therefore, the assignment of xylene to Pregnancy Risk Group D was confirmed. As the BAT value was derived in correlation to the MAK value, Pregnancy Risk Group D applies to the BAT value as well.

**Table:** 

**BAT value (2024)**	**1800 mg Methylhippuric acid/g creatinine**
	Sampling time: end of exposure or end of shift

**MAK value (2019)**	**50 ml/m^3^ ≙ 220 mg/m^3^**
Peak limitation (2001)	Category II, Excursion factor 2
Absorption through the skin (1998)	H
Carcinogenicity	–
Developmental toxicity (1988)	Group D

## Re-evaluation

For xylene (all isomers), BAT values were established for the parameters xylene in blood and methylhippuric acid in urine in correlation to the MAK value (translated in Henschler 1993) in the year 1984; re-evaluation confirmed these values in the year 2000 (translated in Angerer 1995; Angerer and Krämer 2010). In 2015, the BAT value for xylene in blood was withdrawn due to difficulties in sampling (very rapid elimination of xylene from the blood compartment required sampling to take place immediately after the end of exposure) (translated in Jäger et al. 2018).

Since 2016, the Commission accounts for the increased respiratory volume at the workplace, compared to experimental conditions, for substances with MAK values based on systemic effects and which were derived from animal inhalation studies or in humans at rest (Hartwig and MAK Commission 2017). As, taking into account the increased respiratory volume at the workplace, the xylene concentration in the blood at the MAK value of 100 ml xylene/m^3^ was above the threshold concentration for pre-narcotic effects of 2 mg/l derived by MacDonald et al. (2002), the MAK value was lowered to 50 ml xylene/m^3^ (translated in Hartwig and MAK Commission 2022 a). As the BAT value of 2000 mg methylhippuric acid (all isomers)/l urine (Angerer 1995) was derived in correlation to the former MAK value of 100 ml/m^3^, it must be re-evaluated. Moreover, new experimental volunteer studies (Ernstgård et al. 2003; Loizou et al. 1999) and occupational field studies (Jacobson and McLean 2003; Jang et al. 2001; Rajan et al. 2019) have been published since the last evaluation of the BAT value.

### Experimental studies

In a toxicokinetic study with four individuals (two men, two women), Loizou et al. (1999) investigated exposure to an *m*‑xylene concentration of 50 ml/m^3^ without physical exertion over 2 × 12 hours (two half-hour exposure-free times after 4 and 8 hours in each phase). After one hour, the xylene concentration in blood was found to be 0.35 mg/l; after eight hours, this value was 0.6 mg/l. The concentration of methylhippuric acid was determined to be 1100 mg/g creatinine after eight hours.

Ernstgård et al. (2003) conducted an experimental volunteer study with 17 individuals (nine women, eight men), in which each person was exposed to *m*‑xylene at 45 ml/m^3^ over a period of two hours under conditions of light physical activity (50 Watts). At the end of exposure, the *m*‑xylene concentration in blood was found to be 1.23 mg/l for female subjects and 1.17 mg/l for male subjects. The excretion of methylhippuric acid within 24 hours of exposure was determined to be 201 mg for the women and 240 mg for the men.

Tardif et al. (1991) investigated persons who were exposed to xylene (all isomers) without physical exertion. After xylene exposures of 40 ml/m^3^ (5 persons, 7 hours/day, once per week, 3 exposure weeks) or 80 ml/m^3^ (3 persons, 4 hours in one day), urinary methylhippuric acid concentrations of 900 mg/g creatinine and 810 mg/g creatinine, respectively, were determined, whereby sampling took place during the last four hours of exposure.

Šedivec and Flek (1976) exposed four male subjects (respiratory minute volume of 9 l) to 50 ml xylene (all isomers)/m^3^ over a period of eight hours. In the last two hours of exposure, urinary methylhippuric acid concentrations lied in the range of 650.7–1211.3 mg/g creatinine (527.4–2259.1 mg/l).

Jang et al. (1997) investigated six Asians and six Europeans who were exposed to *m*‑xylene at 100 ml/m^3^ over a period of six hours without physical exertion. Statistically, the mean *m*‑methylhippuric acid excretion in urine was significantly higher for the European subjects (2670 mg/g creatinine) than for the Asian subjects (1860 mg/g creatinine).

Ogata et al. (1970) exposed 23 male Asian subjects to 100 or 200 ml *m*‑xylene/m^3^ over a period of seven hours (respiratory minute volume of 8.6 l). In samples collected during the last four hours of exposure, mean *m*‑methylhippuric acid concentrations were found to be 3140 mg/l and 5790 mg/l urine, respectively.

Table 1 provides an overview of results from experimental volunteer studies with exposure to xylene.

**Tab. 1:** Overview of experimental volunteer studies with exposure to xylene

Study design	Xylenes in air [ml/m^3^]	Xylenes in blood [µg/l]	MHA in urine [mg/g Kreatinin]	Factors used	50-ml/m^3^ equivalent^a)^ [mg/g creatinine]	Literatur
23 ♂ Asians; *m*-xylene, 7 h	100	–	3140 mg/l (during exposure (4–8 h))	ET: 8/7 EL: 0.5/0.25 PA: 2 CR: 0.71	2548	Ogata et al. 1970
	200	–	5790 mg/l (during exposure (4–8 h))		2349	
4 ♂ subjects; *o*-, *m*‐, *p*-xylene, 8 h respiratory minute volume of 9 l	50	–	650.7–1211.3 (during the last 2 h of exposure)	PA: 2	1301–2423	Šedivec and Flek 1976
5 ♂ Europeans; nonsmoker xylene (all isomers), 7 h/d on 3 days (in weeks 1, 3, and 5) without physical exertion	40	676 (during (6.5 h) exposure); 316 (after exposure)	900 (during exposure (3–7 h))	ET: 8/7 EL: 1.25 PA: 2	2571	Tardif et al. 1991
3 ♂ Europeans; nonsmoker xylene (all isomers), 4 h, 1 day without physical exertion	80	1140 (during (3.5 h) exposure); 479 (after exposure)	810 (during exposure (0–4 h))	ET: 8/4 EL: 5/8 PA: 2	2025	
6 Asians, 6 Europeans; *m*-xylene, 6 h without physical exertion	100	Asians: 570 ± 260 (0.5 h post-exposure)	Asians: 1860 ± 740	ET: 8/6 EL: 0.5 PA: 2	Asians: 2480	Jang et al. 1997
		Europeans: 610 ± 170 (0.5 h post-exposure)	Europeans: 2670 ± 570		Europeans: 3560	
4 subjects (2 ♂; 2 ♀); *m*-xylene, 2 × 12 h with a 12‑h break (additional 0.5‑h breaks after 4 and 8 h)	50 (51 ± 3)	600 (sampling after 8 h of exposure)	≈ 1100 (sampling after 8 h of exposure)	PA: 2	2200	Loizou et al. 1999
17 subjects (9 ♀; 8 ♂); *m*‑xylene, 2 h physical activity (50 W)	45	1200 (after exposure)	212 mg/24 h	–	–	Ernstgård et al. 2003

^a)^ For an 8-hour exposure with physical exertion. Determinants such as a lack of physical activity, a missing creatinine reference, and shorter exposure times are accounted for by factors.

CR: creatinine reference; EL: exposure level; ET: exposure time; h: hour; MHA: methylhippuric acid; PA: physical activity

### Occupational field studies

Angerer and Krämer (2010) provide a detailed overview of occupational field studies, published up to the year 2000, in which persons with occupational exposure to xylene were examined.

Jang et al. (2001) investigated 20 male metal-processing workers with exposure to ethylbenzene and xylene. Personal air measurements were performed every workday for a week and urine samples were collected at the end of each shift. The mean xylene concentration in the air was 12.77 ml/m^3^ (range: 2.5–61.6 ml/m^3^). The investigation yielded a statistically significant correlation between the xylene concentration in the air and methylhippuric acid (all isomers) concentrations in the urine (r^2^ = 0.503). In this study, external exposure at the level of the MAK value of 50 ml/m^3^ corresponded to a urinary methylhippuric acid concentration of 1011 mg/g creatinine. In the same study, a physiologically based pharmacokinetic (PBPK) model was used to take the influence of combined exposure to xylene and ethylbenzene into account. At an exposure level of 100 ml xylene/m^3^ and simultaneous exposure to 25 ml ethylbenzene/m^3^, the model calculated a methylhippuric acid concentration of 1550 mg/g creatinine.

Jacobson and McLean (2003) investigated 20 individuals (three varnishers, eleven printers, two painters, three automobile painters, one laboratory worker) with occupational xylene exposure. The mean xylene concentration in the air was 3.4 ± 3.6 ml/m^3^ (range: 0.0–14.4 ml/m^3^). Concentrations of methylhippuric acid were determined in urine samples before and after each shift. A correlation was found between total xylene concentrations in the air and creatinine-adjusted methylhippuric acid (all isomers) concentrations in workers’ urine samples (r^2^ = 0.579). Similar findings resulted from the consideration of the individual isomers. An external exposure at the level of the MAK value of 50 ml xylene/m^3^ would correspond to a urinary methylhippuric acid concentration of 735 mg/g creatinine.

Rajan et al. (2019) investigated 45 urine samples from individuals with varying levels of occupational exposure to xylene. Group 1 was comprised of five medical technicians with a 50‑hour workweek, Group 2 of medical technicians with a 30‑hour workweek, and Group 3 of 15 painters; Group 4 was a control collective without exposure to xylene. The mean urinary *o*-, *m*-methylhippuric acid concentrations were 240.0 ± 54.77 mg/g creatinine for Group 1, 33.0 ± 8.23 mg/g creatinine for Group 2, 25.3 ± 13.02 mg/g creatinine for Group 3, and 0.2 ± 0.12 mg/g creatinine for the control group (Group 4). The health status of each study participant was surveyed using a questionnaire, whereby study participants described the occurrence of eye, nose and throat irritation, dizziness, fatigue, stomach pain, loss of appetite, unsteady gait and dermatitis. This is the only study in which, after exposure to xylene, symptoms were reported at such low methylhippuric acid concentrations. As this study did not publish any air measurements, it cannot be used for the derivation of the BAT value in correlation to the MAK value of 50 ml xylene/m^3^.

Table 2 provides an overview of results from occupational field studies with exposure to xylene and the MAK‑value equivalents calculated therefrom.

**Tab. 2:** Overview of field studies with exposure to xylene

Subjects/occupation	Xylenes in air [ml/m^3^]^a)^	Xylenes in blood [µg/l]	Calculated 50-ml xylene/m^3^ equivalent [µg xylene/l]	*o*-, *m*-, *p*-MHA in urine [mg/g creatinine]	Equation	Calculated 50-ml xylene/m^3^ equivalent (*o*-, *m*-, *p*-MHA)	Reference
15 painters (14 ♂, 1 ♀); *o*‐, *m*‐, *p*-xylene, ethylbenzene	range: 3–70		–				Engström et al. 1978
	≤ 20	0.07–0.42					
	21–40	0.33–0.62					
	> 40	0.31–1.36					
	50		–	665 (only *m*‐, *p*-MHA)	not given	764 mg/g creatinine (*o*‐, *m*‐, *p*-MHA)	
35 ♂ varnishers (6 different work places); *o*‐, *m*‐, *p*-xylene, ethylbenzene	mean: 12,8 4.6–19.4^b)^	mean: 164 58–226^b)^	641	411 mg/l 286–588 mg/l^b)^	not given	≈ 1000 mg/l (≙ 715 mg/g creatinine)	Angerer and Wulf 1985
14 workers (degreasing of metal components)	mean: 75 range: 25–112	mean: 1370 range: 340–3100 y = 0.0189x – 0.0863; r = 0.66	859	mean: 1195 mg/l range: 501–2458 mg/l	y = 14.43x – 82.66; r = 0.73	639 mg/l (≙ 456 mg/g creatinine)	Zinser et al. 1985
121 varnishers (♂)^c)^	mean: 3.8 max: 61	not analysed	–	mean: 58 max: 1201	y = 17.8x + 3.8; r = 0.80	894 mg/l (≙ 639 mg/g creatinine)	Kawai et al. 1991
51 varnishers (♂)^c)^	mean: 8 max: 27	y = 11x – 19; r = 0.88	531	–	y = 15.6x + 49; r = 0.72	829 mg/l (≙ 592 mg/g creatinine)	Kawai et al. 1992
175 (107 ♂, 68 ♀), printers, plastics processing^c)^	mean: 14 max: 175	not analysed	–	–	y = 13x + 31; r = 0.82	681 mg/l (≙ 568 mg/g creatinine)	Inoue et al. 1993
233 (122 ♂, 111 ♀), printers, varnishers, plastics processing^c)^	mean: 3 max: 103	not analysed	–	–	y = 14.4x + 37	757 mg/l (≙ 630 mg/g creatinine)	Huang et al. 1994
38 workers^c)^	mean: 14	not analysed	–	320	y = 20x + 0; r = 0.96	1000 mg/l (≙ 714 mg/g creatinine)	Ogata et al. 1995
13 ♂ lacquer-production workers	mean: 29 range: 5–58	mean: 380 range: 63–715 y = 7.5x + 164; r = 0.60	539	mean: 1221 mg/l range: 194–2333 mg/l	y = 32.5x + 290; r = 0.78	1915 mg/l (≙ 1367 mg/g creatinine)	Krämer et al. 1999
10 ♂ varnishers	mean: 8 range: 3–21	mean: 120 range: 24–308 y = 15x + 4; r = 0.75	754	mean: 450 mg/l range: 65–1633 mg/l	y = 73x – 116; r = 0.87	3534 mg/l (≙ 2525 mg/g creatinine)	
20 ♂ workers, metal processing^c)^	mean: 12.77 range: 2.5–61.6	not analysed	–	–	y = 19x + 61; r = 0.71	1011 mg/g creatinine	Jang et al. 2001
20 (18 ♂, 2 ♀) varnishers, painters, printers, automobile painters, laboratory workers	mean: 3.36 ± 3.63 range: 0.03–14.44	not analysed	–	mean: 57.2 ± 60.7 range: 0.2–248.9	y = 12.733x + 14.439; r^2^ = 0.579	651 mg/g creatinine	Jacobson and McLean 2003

^a)^ personal sampling, 8 hours

^b)^ mean values at 6 different work places

^c)^ Asian collective

max: maximum; MHA: methylhippuric acid

## Re-evaluation of the BAT value

Both occupational field studies and experimental volunteer studies are available for the re-evaluation of the BAT value for xylene.

It is important to note that, due to a divergent composition of enzymes and a resulting lower methylhippuric acid excretion in urine compared with Europeans, data with Asian study collectives can only be used to a limited extent for the evaluation of a BAT value (Angerer and Krämer 2010; Jang et al. 1997).

For exposure at the level of the MAK value of 50 ml/m^3^, the data from European occupational field studies (see Table 2) allow for the calculation of a mean methylhippuric acid level of about 1093 mg/g creatinine (range: 456–2525 mg/g creatinine). In most occupational field studies, however, the concentration of xylene in the air was considerably less than 50 ml/m^3^, such that extrapolation to a higher concentration range is relatively uncertain. Furthermore, combined exposure with ethylbenzene, which is common at many workplaces due to the application of technical xylene or C8‑aromatic mixtures, may lead to a reduced excretion of methylhippuric acid (Engström et al. 1984). The reports from these occupational field studies unfortunately lack information which would enable an estimation of the physical activity of the workers during exposure.

Due to the defined exposure situation, generally at the level of the current MAK value, and the exclusion of dermal absorption from direct skin contact, experimental volunteer studies are suitable for the derivation of a BAT value for xylene. Studies with very short exposure durations (< 4 h) and/or with concentrations of methylhippuric acid given in mg/24 h (Ernstgård et al. 2003) are not considered for derivation. The highly consistent data from the experimental volunteer studies by Tardif et al. (1991), Jang et al. (1997), and Loizou et al. (1999) (see Table 1) yield, for an eight-hour exposure period at the level of the MAK value of 50 ml/m^3^, a mean correlate for the excretion of methylhippuric acid at the end of a shift of 1295 mg/g creatinine (range: 1013–1780 mg/g). This value or range of values is quite consistent with those reported by occupational field studies.

The majority of experimental volunteer studies on xylene were, however, performed without physical exertion. According to the standard procedure of the Commission regarding the consideration of the increased respiratory minute volume at the work place, a correction factor of 2 is used for studies with test persons at rest (translated in Greim 2000; Hartwig and MAK Commission 2017). A study by Gamberale et al. (1978), which investigated the influence of physical activity on xylene absorption, showed that, when using exercise with a bicycle ergometer during half of the exposure time, the absorption of xylene was about twice as high over the entire exposure period than when at rest. The urinary methylhippuric acid concentration was not investigated in this study. The study by Ernstgård et al. (2003) likewise confirms the influence of physical activity on xylene concentrations in blood. At 1.2 mg xylene/l blood, values which were twice as high as those of studies without physical exertion – such as from Tardif et al. (1991), Jang et al. (2001), and Loizou et al (1999) – were determined despite a shorter exposure time (see Table 1). Due to the very short exposure time and the provision of methylhippuric acid concentrations in mg/24 h, this study is, however, not suitable for the derivation of a BAT value.

As the experimental data described here were collected without physical activity, the correction factor of 2 for the consideration of an increased respiratory minute volume in the workplace yields, from experimental volunteer studies and for eight-hour exposure at the level of the MAK value of 50 ml/m^3^, a mean equivalent for methylhippuric acid excretion at the end of a shift of 2590 mg/g creatinine.

In any case, the consistency of the results from occupational field studies and experimental studies without physical exertion indicate that physical exertion at workplaces with xylene exposure did not lead to any relevant increase in respiratory volume, and that the standard factor of 2, in this case, overestimates the actual work performance in the workplaces examined. Furthermore, at workplaces in which combined exposure to ethylbenzene is a regular occurrence, a reduction of methylhippuric acid excretion by about 20% is to be expected (Engström et al. 1984).

Taking into account these limitations as well as the complete picture of all available field and experimental studies, the following rounded mean value is derived as

**a BAT value: 1800 mg methylhippuric acid (all isomers)/g creatinine.**

Sampling should take place at the end of exposure or the end of a shift.

## BAT value and pregnancy 

In 2020, the MAK value for xylene was re-evaluated with regard to developmental toxicity (translated in Hartwig and MAK Commission 2022 b). Critical effects of xylene include acute neurotoxicity in humans as well as in animal experiments. Due to mixed exposures and limited validity, human studies are not suitable for the evaluation of the developmental toxicity of xylene. Xylene was shown not to be teratogenic in animal experiments. In rats, the no observed adverse effect concentration (NOAEC) for reduced foetal weight was found to be 100 ml/m^3^ for *o*‑xylene and technical xylene. The other isomers only trigger this effect at higher concentrations. Moreover, increased incidences of skeletal variations occur with *o*‑xylene at 1000 ml/m^3^ as well as with *m*‑ and *p*‑xylene at 2000 ml/m^3^. The NOAEC for maternal toxicity, in the form of delayed body weight development and reduced feed consumption, was 100 ml/m^3^ for all isomers and 500 ml/m^3^ for technical xylene (Greim 2004; Saillenfait et al. 2003). For the assessment of developmental toxicity, only one study on prenatally exposed Sprague-Dawley rats is available, whereby even the highest concentration of 1589 ml *p*‑xylene/m^3^ yielded no effects when testing acoustic startle responses and locomotor activity in the postnatal phase (translated in Greim 2001; Rosen et al. 1986). As, however, the *o*‑isomer proved to be more potent in terms of reduced body weight compared to the *p*‑isomer (Saillenfait et al. 2003), the *o*‑isomer was not investigated in the study by Rosen et al. (1986), and no comprehensive studies on the developmental toxicity of the various isomers are available, the classification of xylene into Pregnancy Risk Group D is confirmed. Because the BAT value is derived in correlation to the MAK value, Pregnancy Risk Group D is likewise valid for the BAT value.

## Interpretation

Past BAT‑value derivations did not apply a creatinine reference for methylhippuric acid. Several publications have shown that associating methylhippuric acid concentrations with the creatinine content of a sample leads to better correlation of the study results (Engström et al. 1978; Šedivec and Flek 1976). Furthermore, the concentrations reported by publications used for the derivation of the BAT value were primarily creatinine-related. For the conversion of volume-related methylhippuric acid concentrations, a factor of 0.83 is used for collectives with a balanced gender distribution and a correction factor of 0.71 is used for male-dominated collectives (Bader et al. 2020).
